# Indirect regulation of TFPI-2 expression by miR-494 in breast cancer cells

**DOI:** 10.1038/s41598-020-61018-x

**Published:** 2020-03-04

**Authors:** Marianne S. Andresen, Benedicte Stavik, Marit Sletten, Mari Tinholt, Per Morten Sandset, Nina Iversen, Grethe Skretting

**Affiliations:** 1 Department of Haematology, Oslo University Hospital, Box 4950 Nydalen, 0424 Oslo, Norway; 2 Research Institute of Internal Medicine, Oslo University Hospital, Box 4950 Nydalen, 0424 Oslo, Norway; 3 Department of Medical Genetics, Oslo University Hospital, Box 4950 Nydalen, 0424 Oslo, Norway; 4 Institute of Clinical Medicine, University of Oslo, Box 1072 Blindern, 0316 Oslo, Norway

**Keywords:** Molecular biology, miRNAs

## Abstract

TFPI-2 has been shown to be involved in breast cancer pathogenesis by inhibiting extracellular matrix degradation, and low levels are associated with disease progression. As microRNA-494 (miR-494) protects against breast cancer progression, we investigated whether miR-494 is involved in the regulation of TFPI-2 in MCF-7 breast cancer cells. TFPI-2 mRNA and protein levels increased after transfection with miR-494 mimic, and TFPI-2 mRNA and miR-494 levels correlated positively in tumors from breast cancer patients. No specific binding sites for miR-494 in the 3′-untranslated region (UTR) of *TFPI2* were identified; however, miR-494 was predicted *in silico* to bind 3′-UTR of the transcription factors AHR and ELF-1, which have potential binding sites in the *TFPI2* promoter. ELF-1 mRNA was downregulated whereas AHR mRNA levels were upregulated after transfection with miR-494 mimic. Knockdown of ELF-1 and AHR increased and reduced TFPI-2 mRNA levels, respectively. Increased luciferase activity was seen when TFPI-2 promoter constructs containing the potential AHR or ELF-1 binding sites were co-transfected with miR-494 mimic. In conclusion, TFPI-2 mRNA levels were upregulated by miR-494 in MCF-7 breast cancer cells most likely by an indirect association where miR-494 targeted the transcription factors AHR and ELF-1. This association was supported in a breast cancer cohort.

## Introduction

Tissue factor pathway inhibitor (TFPI)-2 is a Kunitz-type serine proteinase inhibitor^1,2^ that has been identified as a potent tumor suppressor^3^. It is synthesized by endothelial cells in the vasculature and the majority of the protein is secreted into the extracellular matrix (ECM)^4^. TFPI-2 inhibits a wide variety of serine proteinases including plasmin and trypsin, which activate several pro-matrix metalloproteinases (MMPs) and diminish ECM degradation, a process important for tumor invasion and metastasis^5,6^. TFPI-2 has been shown to be involved in breast cancer pathogenesis. Breast cancer is the leading cause of cancer associated death in women, mostly due to metastatic progression^7^. Breast cancer is a complex and highly heterogeneous disease^8^ and the molecular mechanisms underlying the disease are poorly understood. *In vitro*, TFPI-2 has been reported to suppress breast cancer cell proliferation and invasion^9^, and reduced TFPI-2 expression due to deviant methylation of CpG islands in the *TFPI2* promoter region in breast cancer cell lines, especially in highly invasive cells^10^, has been demonstrated. We have previously reported that high expression of TFPI-2 was associated with increased metastasis free survival in patients with estrogen receptor (ER) α positive tumors^11^. Low TFPI-2 expression levels have been linked to cancer progression, recurrence, and poor survival in patients with breast cancer and TFPI-2 mRNA levels in malignant breast tumors were demonstrated to be lower than in normal breast tissues^12^. Consequently, TFPI-2 has been proposed as a tumor suppressor and a potential prognostic marker in breast cancer, however, little is known about the regulation of TFPI-2 expression after transcription.

MicroRNAs (miRNAs) are small non-coding RNAs (20–23 nucleotides) that play an important role in the control of gene expression^13^. Imperfect complementarity between miRNA and their targets allows each miRNA to regulate more than 100 targets, while one target can be regulated by several miRNAs^14^. One miRNA can regulate a variety of cellular processes through the regulation of multiple target genes including cellular growth, differentiation, proliferation, angiogenesis and apoptosis^15^. miRNAs function primarily by binding to the 3′-UTR of their target mRNAs and exert post transcriptional gene silencing by degrading mRNA or inhibition of translation. Recently, a number of studies indicate that miRNA can also be involved in activation of gene transcription^16–18^.

miRNAs are often dysregulated in a wide variety of cancers and are thus involved in the development of human carcinogenesis by inhibiting or enhancing the expression of tumor genes^19^. As such, they can act both as oncogenes and as tumor suppressors through a direct interaction with the regulated genes or through an indirect modulation of the regulatory systems within the cell^20^. In breast cancer cells, the miRNA miR-494 has been identified as a tumor suppressor by downregulating the expression levels of various proteins with oncogenic activity. miR-494 has been demonstrated to be downregulated both in breast cancer cell lines and breast cancer tissue^21^. Moreover, miR-494 has been reported to inhibit breast cancer progression, both *in vitro* and *in vivo*. However, the underlying mechanisms of how miR-494 function in breast cancer is still unclear.

In this study we aimed to explore if miR-494 is involved in the regulation of TFPI-2 in breast cancer cells using the breast cancer cell line MCF-7. We found that miR-494 increases TFPI-2 levels, most likely through an indirect mechanism involving regulation of transcription factors with potential binding sites in the *TFPI2* promoter. In addition, a positive correlation between miR-494 and TFPI-2 mRNA expression levels in a clinical breast cancer material was demonstrated.

## Results

### miR-494 mimic increased TFPI-2 mRNA and protein expression

**Figure 1:**
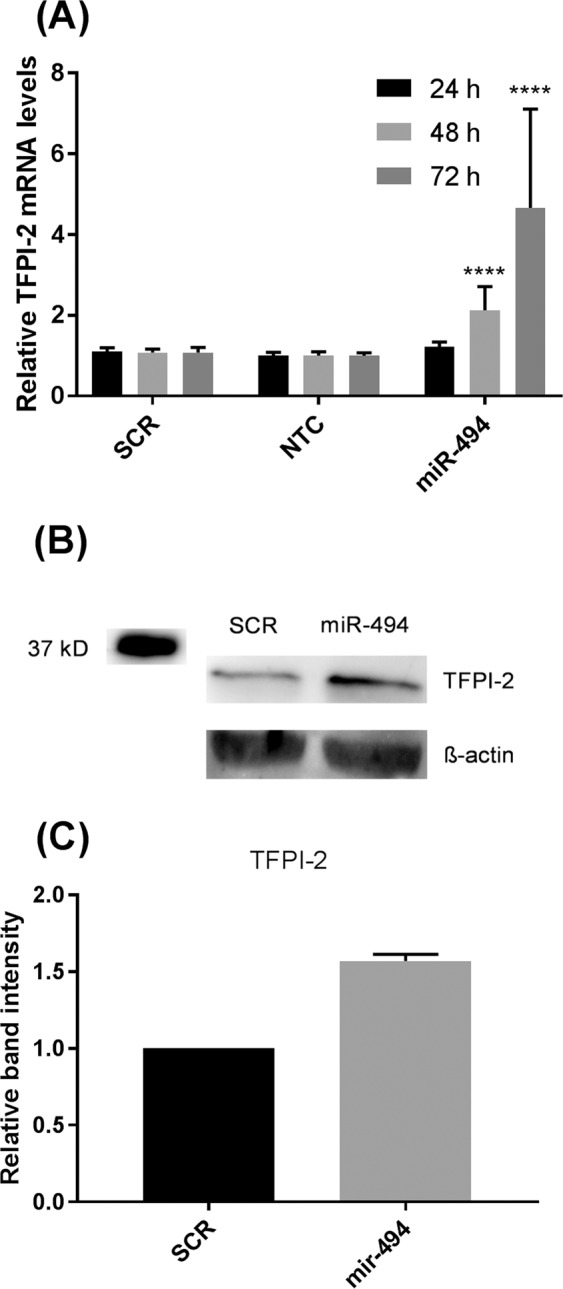
miR-494 mimic increased TFPI-2 mRNA and protein levels. MCF-7 cells were transfected with 10 nM scrambled miRNA (SCR) or miR-494 mimic. Non-transfected cells (NTC) were included for comparison. (**A**) qRT-PCR was used to measure mRNA levels for indicated time points after adjusting for the endogenous control gene levels. The error bars represent standard deviation from at least three independent experiments with three biological parallels (n ≥ 9), relative to SCR transfected cells ****p < 0.0001 as determined by one-way ANOVA. (**B**) Western blots of TFPI-2 protein levels in cell lysates from MCF-7 cells transfected with 10 nM scrambled miRNA (SCR) or miR-494 mimic. TFPI-2 levels were adjusted for β-actin. Full length blots are presented in Supplementary Fig. S1. (**C**) Densitometric analysis of two Western blots (pooled samples from 3 parallels for each Western blot) of TFPI-2 protein levels in cell lysates from MCF-7 cells transfected with 10 nM scrambled miRNA (SCR) or miR-494 mimic. TFPI-2, tissue factor pathway inhibitor-2.

To investigate whether miR-494 could affect the TFPI-2 mRNA and protein expression levels, MCF-7 cells were transfected with miR-494 mimic and harvested after 24, 48 and 72 hours. As shown in Fig. 1A, TFPI-2 mRNA was significantly increased two-fold after 48 hours and more than four-fold after 72 hours. A scrambled miR (SCR) was included as a negative control. Western blot analysis demonstrated increased TFPI-2 protein levels by approximately 50% 48 hours after transfection with the miR-494 mimic (Fig. 1B,C).

### *In silico* search of miR-494 targeting transcription factors with potential binding sites within the *TFPI2* 5′-flanking region

No specific binding sites for miR-494 in the 3′-UTR of *TFPI2* were predicted by the online miRNA prediction programs miRSVR and TargetScan, indicating that the regulation of *TFPI2* by miR-494 might be indirect. We therefore searched for transcription factors that both had the potential to regulate TFPI-2 expression and at the same time could be a target for miR-494. To identify transcription factors with potential binding sites in the *TFPI2* 5′-flanking region we used the program PROMO with a dissimilarity rate cut-off set to ≤ 10%. Potential binding sites for 35 different transcription factors were identified. To test whether these factors were possible targets for miR-494, we analysed the 3′-UTRs of the transcription factors according to miRNA binding scores. The results revealed that the transcription factors AHR, AR, ATF-2, c-Fos, CREB1, EBF1, ELF-1, ELK-1, HOXD9, Ik-1, Jun (AP1), POU2F2, RBP-J kappa, SMAD3, Sp1, STAT1 beta and WT1 had predicted binding sites for miR-494 in their 3′-UTRs. Of these, we selected nine candidate transcription factors (underlined) for further testing.

### miR-494 mimic increases AHR expression, but decreases ELF-1 and SMAD3 expression

**Figure 2:**
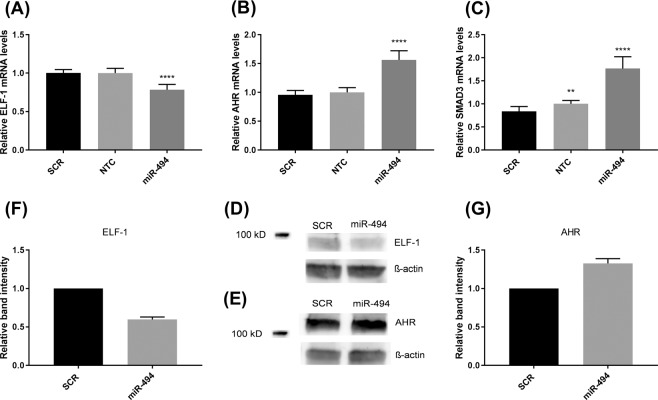
Effect of miR-494 mimic on ELF-1 (**A**), AHR (**B**) and SMAD3 (**C**) transcription factor mRNA levels. MCF-7 cells were transfected with 10 nM scrambled miRNA (SCR) or miR-494 mimic. Non-transfected cells (NTC) were included for comparison. qRT-PCR was used to measure mRNA levels after 48 hours. The results were normalised against the endogenous control gene mRNA levels and presented relative to SCR transfected cells, ****p < 0.0001 as determined by one-way ANOVA. The error bars represent standard deviation from at least three independent experiments with three biological parallels (n ≥ 9). Western blots of ELF-1 (**D**) and AHR (**E**) protein levels in cell lysates from MCF-7 cells transfected with 10 nM scrambled miRNA (SCR) or miR-494 mimic. ELF-1 and AHR levels were adjusted for β-actin. Full length blots are presented in Supplementary Fig. S2A, B. Densitometric analysis of two Western blots (pooled samples from 3 parallels for each Western blot) of ELF-1 (**F**) and AHR (**G**) protein levels in cell lysates from MCF-7 cells transfected with 10 nM scrambled miRNA (SCR) or miR-494 mimic. ELF-1, E74-like factor-1; AHR, aryl-hydrocarbon receptor.

To explore whether miR-494 could affect the mRNA expression of the selected transcription factors, MCF-7 cells were transfected with miR-494 mimic and harvested after 48 hours. Transcription factor mRNA levels were measured by qRT-PCR. E74-like factor 1 (ELF-1) mRNA levels were significantly downregulated by approximately 25%, whereas aryl-hydrocarbon receptor (AHR) and SMAD3 mRNA levels were significantly upregulated by 55% and 75% respectively, compared to SCR controls (Fig. 2A–C). Consistent with the decrease in ELF-1 mRNA levels, Western blot analysis showed decreased ELF-1 protein levels in MCF-7 cells 72 hours after transfection with miR-494 mimic (Fig. 2D,F). We also observed an increase in AHR protein levels 48 hours after transfection with miR-494 mimic (Fig. 2E,G). Only minor differences in the mRNA expression levels of AR, ATF-2, c-Fos, CREB1, Jun (AP1) and Sp1, were found after transfection (data not shown).

### ELF-1 and AHR levels affected TFPI-2 expression

**Figure 3:**
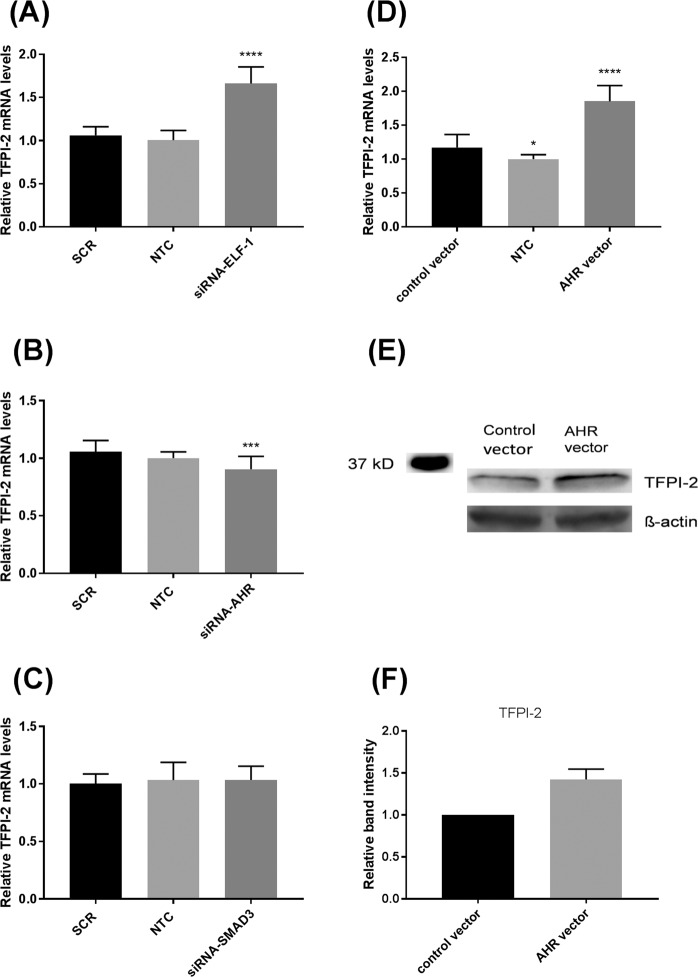
ELF-1 and AHR levels affected TFPI-2 expression. MCF-7 cells were transfected with 10 nM scrambled control (SCR) and siRNA-ELF-1 (**A**), siRNA-AHR (**B**) or siRNA-SMAD3 (**C**) for 24–48 hours. Non-transfected cells (NTC) were included for comparison. The results were normalised against the endogenous control gene mRNA levels and presented relative to SCR transfected cells. The error bars represent standard deviation from at least three independent experiments with three biological parallels (n ≥ 9), ***p < 0.001, ****p < 0.0001 as determined by one-way ANOVA. MCF-7 cells were transfected with empty control vector or AHR expression vector. Non-transfected cells (NTC) were included for comparison. (**D**) qRT-PCR was used to measure mRNA levels after 24 hours. The results were normalised against the endogenous control gene mRNA levels and presented relative to cells transfected with empty vector, ****p < 0.0001 as determined by one-way ANOVA. The error bars represent standard deviation from two independent experiments with three biological parallels (n = 6). (**E**) Western blots of TFPI-2 protein levels in cell lysates from MCF-7 cells transfected with empty control vector or AHR expression vector. TFPI-2 levels were adjusted for β-actin. Full length blots are presented in Supplementary Fig. S3G. (**F**) Densitometric analysis of two Western blots (pooled samples from 3 parallels for each Western blot) of TFPI-2 protein levels in cell lysates from MCF-7 cells transfected with empty control vector or AHR expression vector. ELF-1, E74-like factor-1; AHR, aryl-hydrocarbon receptor; TFPI-2, tissue factor pathway inhibitor-2.

To assess whether the levels of ELF-1, AHR or SMAD3 could influence the *TFPI2* expression the transcription factors were knocked down by transiently transfecting MCF-7 cells with siRNAs targeting the corresponding transcription factors. TFPI-2 mRNA levels were quantified 24–48 hours post transfection. Knockdown of ELF-1 resulted in a significant 65% increase in TFPI-2 mRNA levels compared to the SCR control (Fig. 3A). In contrast, transfection of siRNA targeting AHR reduced the TFPI-2 mRNA level by 10% compared to SCR-transfected cells (Fig. 3B). The efficiency of knockdown on the mRNA levels was 57% and 58% for ELF-1 and AHR, respectively (Supplementary Fig. S3A,B). No effect of SMAD3 knockdown on TFPI-2 mRNA expression level was observed (Fig. 3C.) Knockdown of AHR also resulted in reduced TFPI-2 protein levels by approximately 20% after 48 hours (Supplementary Fig. S3C,D), whereas knockdown of ELF-1 increased TFPI-2 protein levels about 20% (Supplementary Fig. 3E,F). Overexpressing AHR for 24 hours lead to a significant upregulation of TFPI-2 mRNA levels by approximately 70% (Fig. 3D) and increased TFPI-2 protein levels by approximately 40% as demonstrated by Western blot analysis (Fig. 3E,F).

### Association between miR-494 and ELF-1 or AHR in the *TFPI2* 5′-flanking region

**Figure 4:**
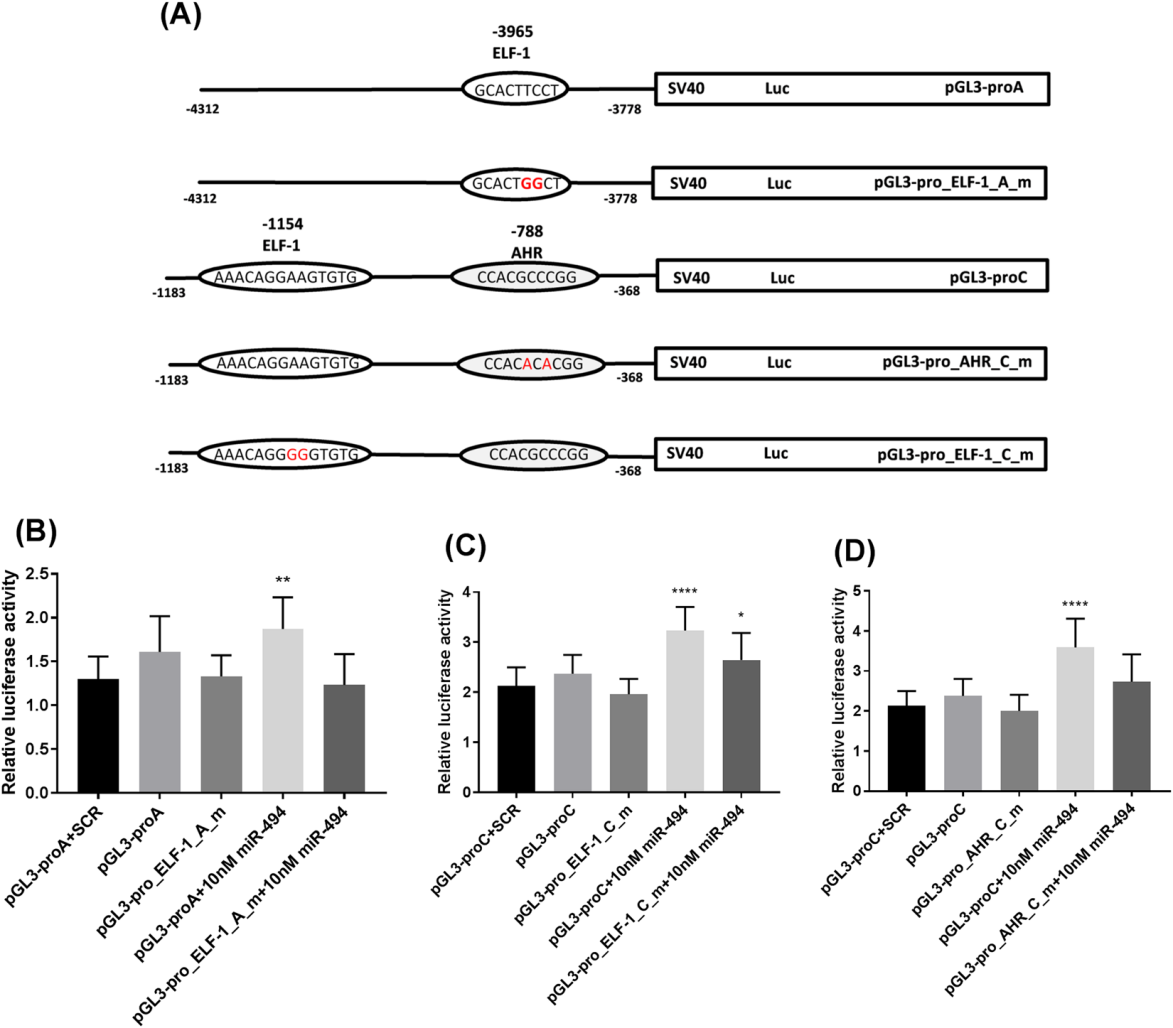
Transcriptional activity in the 5′-upstream region of the *TFPI2* gene. Constructs containing the ELF-1 and AHR binding sites in the 5′-upstream region of the *TFPI2* gene was generated in the luciferase pGL3-promoter vector and were used to create constructs with mutated AHR or ELF-1 sites using site directed mutagenesis (**A**). The cells were co-transfected with miR-494 and constructs of the *TFPI2* 5′-flanking region containing wild type or mutated binding sites for AHR located at −788 (**B**), ELF-1 located at −1154 (**C**) or ELF-1 located at −3965 (**D**). 48 hours after transfection the cells were analyzed for luciferase activity. The luciferase activity was normalized against the renilla luciferase activity. The error bars represent standard deviation from at least three independent experiments (n ≥ 9), *p = 0.0427, **p = 0.0022, ****p < 0.0001 relative to SCR transfected cells as determined by one-way ANOVA. TFPI-2, tissue factor pathway inhibitor-2; ELF-1, E74-like factor-1; AHR, aryl-hydrocarbon receptor.

Two potential binding sites for ELF-1 and one potential binding site for AHR within the *TFPI2* 5′-flanking region were identified in the *in silico* analysis. To examine the interactions of miR-494 with the potential target sites of ELF-1 or AHR within the *TFPI2* 5′-flanking region, five luciferase reporter constructs were generated containing elements of the *TFPI2* 5′-flanking region comprising the wild type or mutated ELF-1 or AHR transcription factor binding sites (Fig. 4A). The first construct contained one binding site for ELF-1 (pGL3-proA). A mutated ELF-1 sequence was introduced by site-directed mutagenesis to generate a second construct where the binding site was disrupted (pGL3-pro_ELF-1_A_m). The third construct contained one binding site for AHR and one binding site for ELF-1 (pGL3-proC). From this, two additional constructs were made by site-directed mutagenesis, one with mutated AHR binding site (pGL3-pro_AHR_C_m), and one with mutated ELF-1 binding site (pGL3-pro_ELF-1_C_m). The constructs were transfected into MCF-7 cells alone or in combination with miR-494 mimic or scrambled control. Co-transfection of the construct containing one potential ELF-1 binding site at position -3965 (pGL3-proA) with miR-494 mimic increased the luciferase activity significantly of approximately 40% compared to when co-transfected with the negative control (SCR). No effect on luciferase activity were seen when the mutated construct pGL3-pro_ELF-1_A_m were co-transfected with the miR-494 mimic (Fig. 4B). A significant increase in luciferase activity of 60–70% was also obtained when the construct containing one potential binding site for AHR at position −788 and one potential binding site for ELF-1 at position −1154 (pGL3-proC) was co-transfected with the miR-494 mimic compared to the scrambled control (Fig. 4C,D). No effects on luciferase activity were seen when the mutated construct pGL3-pro_AHR_C_m were co-transfected with the miR-494 mimic (Fig. 4D), however, a small increase in the luciferase activity was obtained when the pGL3-pro_ELF-1_C_m construct was co-transfected with the miR-494 mimic compared to the negative control (SCR) (Fig. 4C). Together these results indicated an association between AHR and ELF-1 with the miR-494.

### miR-494 expression correlated to TFPI-2 mRNA levels in clinical material

**Figure 5:**
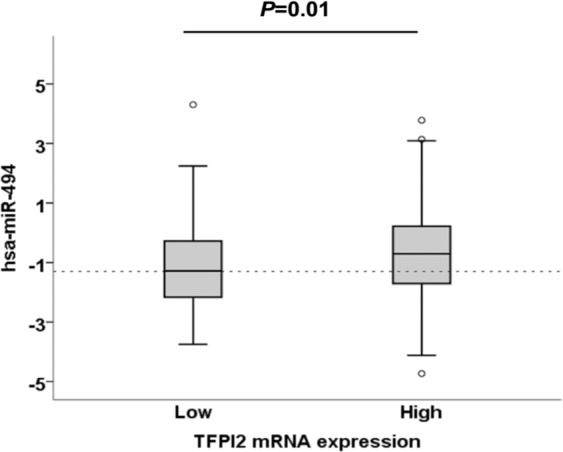
Box and whiskers plot of hsa-miR-494 expression according to low TFPI-2 mRNA expression (below median) and high TFPI-2 mRNA expression (above median) in tumors of 358 breast cancer patients. Log2 transformed expression values are shown. The independent samples *t*-test was applied to compare the mean miR expression between the two groups. *P*-values are indicated as determined by Students *t*-test. TFPI-2, tissue factor pathway inhibitor-2.

To investigate whether the association between miR-494 and TFPI-2 could be relevant in a clinical setting, we examined the relationship between TFPI-2 mRNA and miR-494 levels in tumors from breast cancer patients. miR-494 levels were dichotomized according to TFPI-2 mRNA levels below (low) or above (high) the median and we found that the miR-494 levels were significantly increased in patients with high TFPI-2 mRNA expression compared to patients with low TFPI-2 mRNA expression (*P* = 0.001) (Fig. 5). Correspondingly, we found a positive correlation (r = 0.16, *P* = 0.002) between miR-494 levels and TFPI-2 mRNA levels.

## Discussion

Differential regulation of TFPI-2 has been found in a variety of diseases, including breast cancer, where it has been shown to have a role in tumor suppression^22^. miRNAs are reported to have a consequential effect in breast cancer development and has been shown to have both oncogenic and tumor suppressive roles^23,24^. In the present study we examined the effect of miR-494 on TFPI-2 expression and found increased TFPI-2 levels when miR-494 was overexpressed in the breast cancer cell line MCF-7. No specific binding sites for miR-494 in the 3′-UTR of the *TFPI2* gene were identified, indicating that the association between miR-494 and *TFPI2* was independent of a classical 3′-UTR binding site and might therefore be indirect. Although it is widely recognized that miRNAs mainly target the 3′-UTR of mRNA transcripts leading to inhibition of translation or mRNA degradation, it has lately been reported that miRNAs are able to activate gene transcription by targeting transcription factors that regulate gene transcription^25^. To explore an indirect association between miR-494 and *TFPI2*, transcription factors containing both a potential target sequence for miR-494 in their 3′-UTR and also could potentially bind to the *TFPI2* promoter, were identified by *in silico* search. Of the selected candidates we found that both AHR and ELF-1 expression was affected when the miR-494 mimic was overexpressed in the MCF-7 cells. Increased mRNA and protein levels of AHR were observed, while miR-494 overexpression resulted in reduced ELF-1 mRNA and protein levels.

The *TFPI2* 5′-flanking region contains one potential binding site for AHR and two potential binding sites for ELF-1. We therefore used overexpression and knockdown studies to explore whether AHR and ELF-1 could regulate the TFPI-2 level. Overexpression of AHR and knockdown of ELF-1 both resulted in increased TFPI-2 expression, whereas it was reduced by knockdown of AHR. Using the luciferase reporter gene approach, we observed increased luciferase activity when the construct containing the binding site for AHR in the *TFPI2* 5′-flanking region were co-transfected with the miR-494 mimic, an effect that was abolished when the AHR binding site was disrupted. This indicates that AHR might be an enhancer in this system by binding to this site in the *TFPI2* promoter as a result of miR-494 overexpression and thereby induce the transcription of the gene. AHR is short for aryl hydrocarbon receptor and it is a transcriptional regulator that plays an important role in many biological processes and disease conditions including cancer^26^. It is an intracellular receptor that is activated by a wide range of natural and synthetic molecules, such as exogenous aromatic hydrocarbons and also by endogenous ligands^27^. Upon ligand binding, the receptor heterodimerizes with the AHR nuclear translocator (ARNT) forming a functional AHR-ARNT heterodimer that translocate to the nucleus, and activates target genes^28^. Previous studies have demonstrated that knockout mice deficient in the AHR affected liver development, poor fertility and weight loss^29,30^. This suggests that AHR regulates constitutive functions in the absence of an exogenous ligand. It is suggested that AHR plays a role in cancer progression^31^, while others have shown that under certain circumstances AHR can oppose tumor aggression^32,33^. We demonstrated that AHR enhanced the *TFPI2* transcription indicating that AHR can promote an anti-tumorigenic function. AHR has been reported to exhibit growth inhibitory effects in breast cancer cells in the absence of an exogenous ligand^34^. AHR is positively correlated to overall survival in ER positive tumors supporting a tumor suppressor function^35^. Moreover, co-expression analyses of the interaction between miR-494 and AHR in cancers from brain, breast, prostate and thyroid carcinoma are found to be positively correlated^36^. Similar results as for AHR were obtained for ELF-1 when the two luciferase constructs containing the binding sites for ELF-1 in the *TFPI2* 5′-flanking region were co-transfected with the miR-494 mimic. This suggested reduced binding of ELF-1 to these sites in the *TFPI2* promoter as a result of overexpressing miR-494 and thereby increasing the transcription of the gene. When the ELF-1 sites were mutated, however, the obtained luciferase activity was still reduced when co-transfecting with the miR-494 mimic compared to the wild type. This indicated that ELF-1 is involved in the regulation of TFPI-2 by miR-494, but the specific contribution of the ELF-1 sites investigated here is not fully conclusive. We cannot rule out that an effect on the luciferase activity is due to the non-mutated binding site. Especially, the role of the ELF-1 site located −1154 is somewhat unclear since this construct also contains the AHR wild type binding site. However, we did see a decrease in the luciferase activity with both the pGL3-pro_ELF-1_C_m and pGL3-pro AHR_C_m constructs indicating that both the AHR and the ELF-1 binding sites on the pGL3-proC construct are likely to be important. ELF-1 belongs to the ETS transcription factor family and is mainly located in the nucleus^37^. It is found in tumor cells and is highly expressed in MCF-7 cells^38^. Previous studies indicated both oncogenic and tumor suppressive roles for ELF-1^39–41^. Our results demonstrated that ELF-1 was downregulated and that TFPI-2 mRNA was upregulated by miR-494 in MCF-7 cells suggesting an oncogenic function for ELF-1. Although both AHR and ELF-1 appears to be involved in regulating TFPI-2 expression by miR-494, we cannot rule out that some of the other transcription factors that were not tested also can be involved in this system.

miR-494 is known to play a role in different types of cancer^42^. As miRNAs are tissue and cancer specific they have different functions in different tissues. In most human cancers miR-494 acts anti-tumorigenic^43–45^. We found a positive correlation between miR-494 levels and TFPI-2 mRNA levels in tumors from breast cancer patients supporting a tumor suppressive role. Previous studies have also pointed out that miR-494 acts as a tumor suppressor in breast cancer. PAK1, an oncogene in breast cancer, was identified as a target of miR-494 and thereby inhibiting breast cancer proliferation, colony-formation and cell motility^21^. Additionally, miR-494 suppresses the progression of breast cancer through the Wnt/β-catenin signaling pathway^46^. Overexpression of miR-494 in breast cancer cells implanted into a mice model significantly reduced the number of primary tumors formed and completely abolished tissue invasion^21^. An important tumor suppressor function of TFPI- 2 is the impairment of ECM degradation, and this activity might contribute to the anti-invading effect of miR-494 on breast cancer cells *in vivo*.

In conclusion, we demonstrated that TFPI-2 levels were upregulated by miR-494 in the breast cancer cell line MCF-7 most likely by an indirect regulation involving the association between miR-494 and the transcription factors AHR and ELF-1. We suggest that these two factors might be direct targets for miR-494 and that both AHR and ELF-1 can bind to the *TFPI2* 5′-flanking region, thereby influencing the expression of *TFPI2* and thereby contribute to the tumor suppressor role of miR-494 in breast cancer.

## Methods

### Transfections

The human mammary epithelial adenocarcinoma cell line MCF-7 was obtained and cultured as previously described^47^. For transient knockdown of AHR or ELF-1, MCF-7 cells were transfected with siRNA against ELF-1 or AHR (Trilencer-27 Human siRNA oligonucleotides against ELF-1 (ID 1997) SR301385, siRNA-ELF-1 (siRNA-ELF-1) and # 2 (Supplementary Fig. S3A) and AHR (ID 196) SR300136 (ORIGENE, Rockville, MD, USA)) as previously reported^11^. For overexpression of AHR, MCF-7 cells were transfected with AHR (NM_1621) Human Tagged ORF clone RC209832 or pCMV6-XL5 empty control vector (both from ORIGENE, Rockville, MD, USA). mirVana® hsa-miR-494-3p miRNA mimic MC12409 and miRNA mimic Negative Control #1 (10 nM) were transfected into cells using Lipofectamine® RNAiMAX (all from Thermo Fisher Scientific) following the manufacturer’s procedure.

### Quantitative RT-PCR

Total RNA extraction, RNA concentration, and cDNA synthesis were performed as previously described^11^. Quantitative RT-PCR was performed using TFPI-2 (Hs04334126_m1), AHR (Hs00169233_m1), ELF-1 (Hs01111177_m1), and phosphomannomutase 1 (PMM1) (Hs00160195_m1) Taqman assays as reported in^11^.

### Western blot

MCF-7 cells were harvested and proteins (30–40 µg) were subjected to Western blotting as previously described (Andresen *et al*.^11^), using the primary antibodies AHR (sc-133088), ELF-1 (sc-133096), (both from Santa Cruz Biotechnology, Dallas, Texas, USA), TFPI-2 (ab186747, Abcam, Cambridge, UK) or ß-actin (#4967, Cell Signaling, Danvers, MA, USA).

### *In silico* analysis

Prediction of binding sites for transcription factors in the *TFPI2* 5′-flanking region between +20 to −4760 was achieved using the online prediction tool PROMO version 3.0.2 database, using the TRANSFAC version 8.3^48,49^, with dissimilarity rate cut-off set to ≤10%. Predicted binding sites for miR-494 in the 3′-UTR of *TFPI2* and transcription factors were identified using miRSVR (http://www.microrna.org/microrna/home.do) and TargetScan (http://www.targetscan.org).

### Plasmid constructs

Two regions upstream of the *TFPI2* gene spanning -4312 to -3778 and -1183 to -368 were amplified from human genomic DNA isolated from whole blood with the primers: forward 5′-TCT-GCT-TTA-AGG-TCT-TGG-TAT-GGT-3′ and reverse: 5′-TCC-TGG-AGA-CTA-CTT-TGA-CGT-3′, and forward 5′-ATT-GAT-GCA-GTG-ACC-TGG-GC-3′ and reverse 5′-AGC-CGG-AAT-CCA-CCT-CTT-GA-3′, respectively. The PCR was performed with the AmpliTaq Gold DNA Polymerase using 95 °C for 10 minutes, 30 cycles of 95 °C for 30 seconds, 50 °C for 30 seconds and 72 °C for 1 minute completed with a final extension step at 72 °C for 7 minutes. The PCR fragments were cloned into the pGL3-Promoter vector containing the Simian vacuolating virus 40 (SV40) promoter and luciferase cDNA (Promega (Madison, WI, USA). The pGL3-Promoter-TFPI-2 constructs were used as templates to generate three luciferase constructs with mutated binding sites for aryl-hydrocarbon receptor (AHR) and E74-like factor-1 (ELF-1) using the Kit QuikChange II Site-Directed Mutagenesis (Agilent, Santa Clara, CA, USA). The mutations were verified by sequencing. The primers used for mutated AHR binding site were forward 5′-GGT-GCC-CGC-CAC-CAC-ACA-CGG-CTA-ATT-TTT-TGT-G-3′ and reverse 5′-CAC-AAA-AAA-TTA-GCC-GTG-TGT-GGT-GGC-GGG-CAC-C-3′. The primers used for mutated ELF-1 binding sites were forward 5′-GTT-ATC-TCT-AGC-CTT-GCA-CTG-GCT-GTT-GAC-TTT-GGA-CAA-AGG-3′ and reverse 5′-CCT-TTG-TCC-AAA-GTC-AAC-AGC-CAG-TGC-AAG-GCT-AGA-GAT-AAC-3′; forward 5′-CTG-GGC-GTA-TCT-CAA-AAA-CAG-GGG-GTG-TGA-GAA-AGA-AAT-ACT-ACT-ACA-C-3′ and reverse 5′-GTG-TAG-TAT-TTC-TTT-CTC-ACA-CCC-CCT-GTT-TTT-GAG-AA-CGC-CCA-G-3′.

### Luciferase reporter assays

MCF-7 cells (7 × 10^4^) were seeded in 500 µL DMEM with FBS in 24-well plates and grown for 48 hours. Cells were co-transfected with 0.5 µg of each luciferase construct with or without 10 nM hsa-miR-494-3p miRNA mimic or Negative Control #1 using 1.5 µL Lipofectamine 2000 (Thermo Fisher Scientific) following the manufacturer’s instructions. The cells were in addition co-transfected with pRL-SV40 vector (5 ng) expressing Renilla luciferase DNA (hRluc). Medium was replaced four hours post transfection. After 48 hours, cells were washed in Phosphate-buffered saline (PBS) (GE Healthcare Life Sciences, Pittsburgh, PA, USA) and lysed with 100 µL of passive lysis buffer (1×), before the luciferase activity was quantified in a Glomax®−96 Luminometer (Promega) using the Dual-Luciferase Reporter Assay System (Promega, Madison, WI, USA). Values were corrected for Renilla internal control activity. The luciferase activity from unstimulated cells transfected with the pGL3-promoter was arbitrarily defined as 1 and the miR transfected cells were adjusted accordingly.

### Clinical material

The clinical material was obtained from 358 breast cancer patients with inclusion criteria as previously described^50^. The ER status was known for 273 of the patients (218 (80%) ER positive and 55 (20%) ER negative). Total RNA was isolated from the tumor material and verified by Bioanalyzer, before genome-wide detection of miRNAs and mRNAs was conducted with Agilent microarrays, as previously reported^51^. All experiments were performed in accordance with relevant guidelines and regulations. The study protocol was approved by the Norwegian southeastern Regional Committee for Medical and Health Research Ethics (approval number 1.2007.1125 and 429–04148). Written informed consent was obtained from all participants.

### Statistical analysis

Students *t*-test or one-way ANOVA (Bonferroni corrected tests) was used to calculate statistical significant differences between two or more groups, respectively, in GraphPad Prism (GraphPad Software Inc., San Diego, CA). Spearman correlation was used for analysis of the breast cancer cohort data. Data are presented as mean + SD, and a *P*- value < 0.05 was considered significant.

## Supplementary information

Supplementary information.

## Data Availability

The datasets analysed during the current study are available from the corresponding author on reasonable request.
