# Supplementary material for: Indirect regulation of TFPI-2 expression by miR-494 in breast cancer cells
Source: Sci Rep. 2020 Mar 4;10:4036. doi: 10.1038/s41598-020-61018-x (PMC7055239; doi:10.1038/s41598-020-61018-x)
Supplement: Supplementary file 1 — Supplementary information. [file 41598_2020_61018_MOESM1_ESM.pdf]

Indirect regulation of TFPI-2 expression by miR-494 in breast cancer cells

Marianne S. Andresen[1,2], Benedicte Stavik[1,2], Marit Sletten[3], Mari Tinholt[1,3], Per Morten Sandset[1,2,4], Nina Iversen[3], Grethe Skretting[1,2]

[1]Department of Haematology, [2]Research Institute of Internal Medicine, and

[3]Department of Medical Genetics, Oslo University Hospital, Box 4950 Nydalen, 0424 Oslo, Norway.

[4]Institute of Clinical Medicine, University of Oslo, Box 1072 Blindern, 0316 Oslo, Norway.

Supplementary Figure S1 Western blots of TFPI-2 protein levels in cell lysates from MCF-7 cells transfected with 10 nM scrambled miRNA (SCR) or miR-494 mimic. TFPI-2 levels were adjusted for β-actin. Full length blots are presented. TFPI-2, Tissue factor pathway inhibitor-2.

Supplementary Figure S1

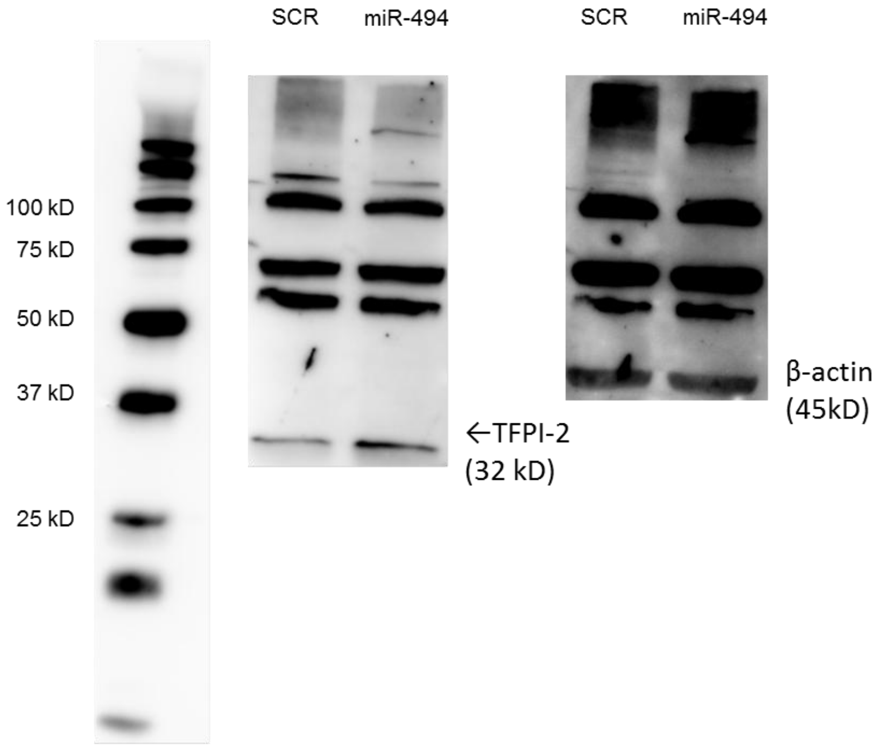

Supplementary Figure S2 Western blots of ELF-1 (**A**) and AHR (**B**) protein levels in cell lysates from MCF-7 cells transfected with 10 nM scrambled miRNA (SCR) or miR-494 mimic. ELF-1 and AHR levels were adjusted for β-actin. Full length blots are presented. ELF-1, E74-like factor-1; AHR, aryl-hydrocarbon receptor.

Supplementary Figure S2

(**A**)

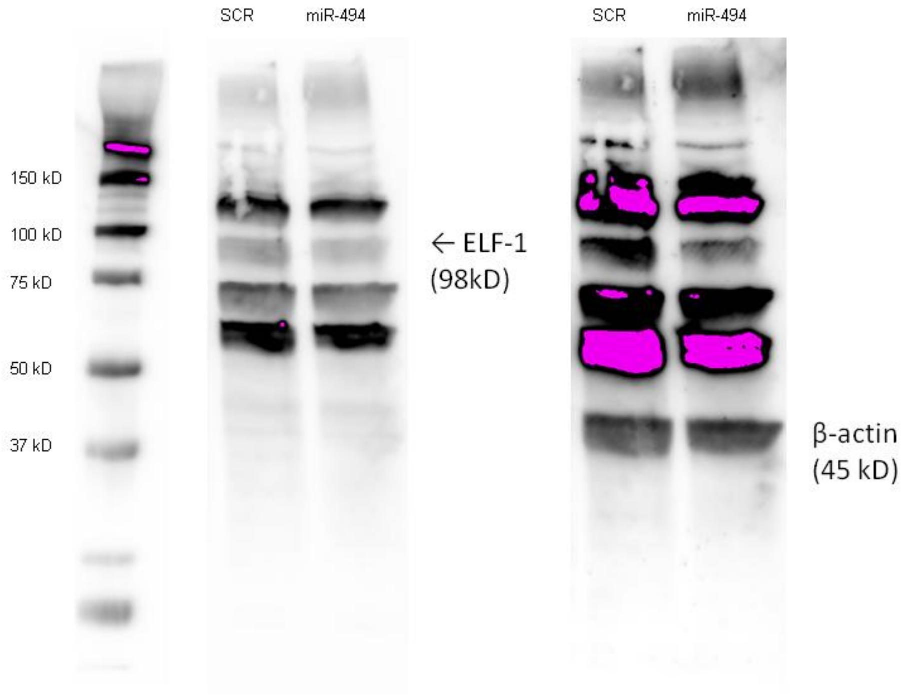

(**B**)

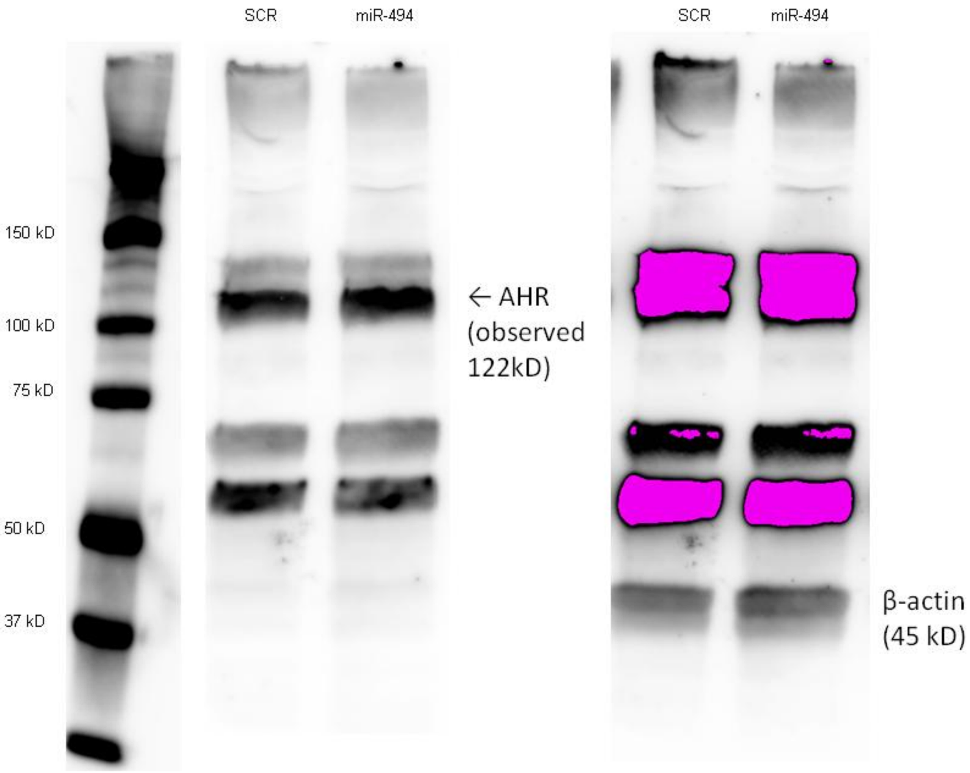
SCR
miR-494
SCR
miR-494
150 kD
100 kD
75 kD
50 kD
37 kD
← AHR (observed 122kD)
β-actin (45 kD)

Supplementary Figure S3 ELF-1 and AHR levels affected TFPI-2 expression. MCF-7 cells were transfected with 10 nM scrambled control (SCR) and siRNA-ELF-1or siRNA-ELF-1, # 2 (**A**) or siRNA-AHR (**B**) for 24-48 hours. Non-transfected cells (NTC) were included for comparison. The results were normalised against the endogenous control gene mRNA levels and presented relative to SCR transfected cells. The error bars represent standard deviation from at least three independent experiments with three biological parallels ($n \geq 9$), ***$p < 0.001$, ****$p < 0.0001$ as determined by one-way ANOVA. For siRNA ELF-1, # 2, the results are presented relative to NTC cells. The error bars represent standard deviation from two independent experiments with three biological parallels ($n = 6$), ****$p < 0.0001$ as determined by Students *t*-test. (**C)** Western blot of TFPI-2 protein levels in cell lysates from MCF-7 cells transfected with 10 nM scrambled control (SCR) or siRNA-AHR for 48 hours. TFPI-2 levels were adjusted for β-actin. (**D)** Densitometric analysis of two Western blots (pooled samples from 3 parallels for each Western blot) of TFPI-2 protein levels in cell lysates from MCF-7 cells transfected with 10 nM scrambled control (SCR) or siRNA-AHR. (**E)** Western blot of TFPI-2 protein levels in cell lysates from MCF-7 cells transfected with 10 nM scrambled control (SCR) or siRNA-ELF-1 for 48 hours. TFPI-2 levels were adjusted for β-actin. (**F)** Densitometric analysis of Western blot (pooled samples from 3 parallels) of TFPI-2 protein levels in cell lysates from MCF-7 cells transfected with 10 nM scrambled control (SCR) or siRNA-ELF-1. (**G**) Western blots of TFPI-2 protein levels in cell lysates from MCF-7 cells transfected with empty control vector or AHR expression vector. TFPI-2 levels were adjusted for β-actin. Full length blots are presented. ELF-1, E74-like factor-1; AHR, aryl-hydrocarbon receptor; Tissue factor pathway inhibitor-2.

Supplementary Figure S3

(**A**)

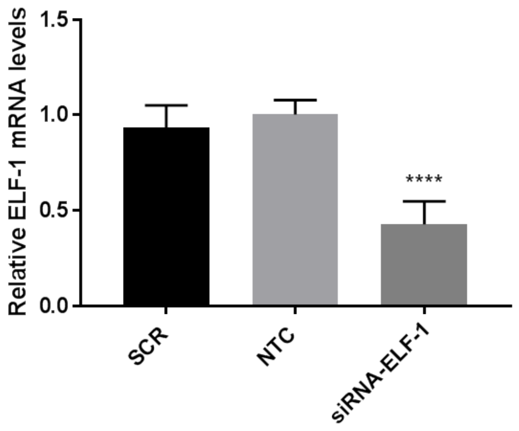

siRNA ELF-1, # 2.

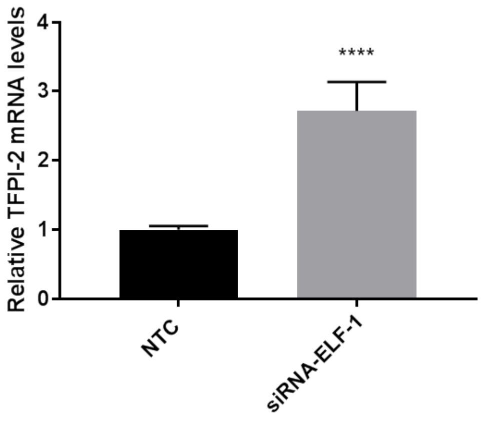

siRNA ELF-1, # 2.

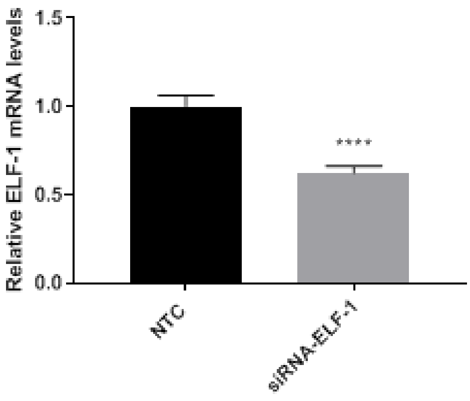

(B)

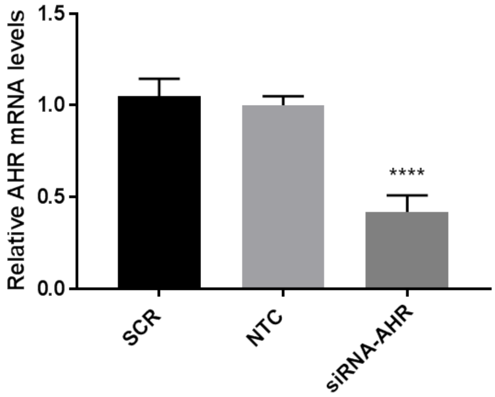

(C)

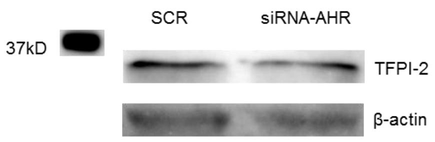

(**D**)

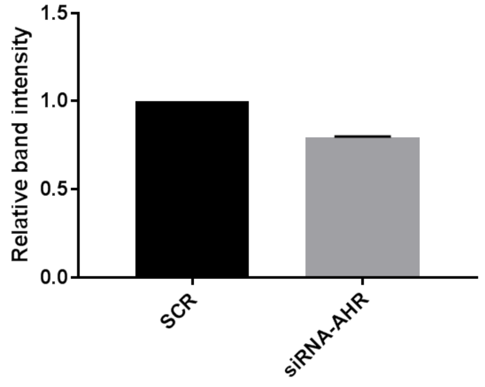

1.5
1.0
0.5
0.0
Relative band intensity
SCR
siRNA-AHR

(**E**)

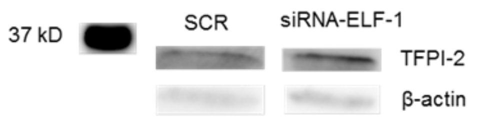

37 kD
SCR
siRNA-ELF-1
TFPI-2
β-actin

(**F**)

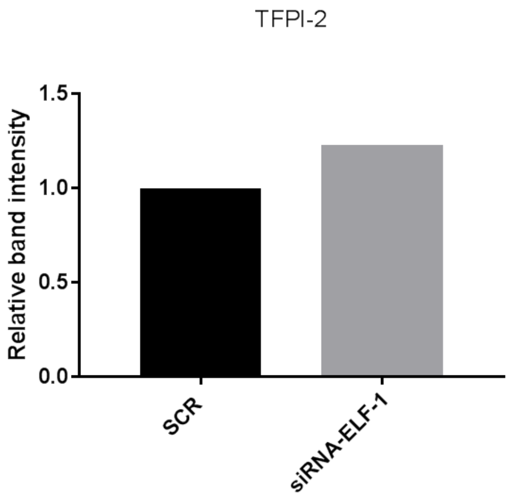

TFPI-2
1.5
1.0
0.5
0.0
Relative band intensity
SCR
siRNA-ELF-1

(G)

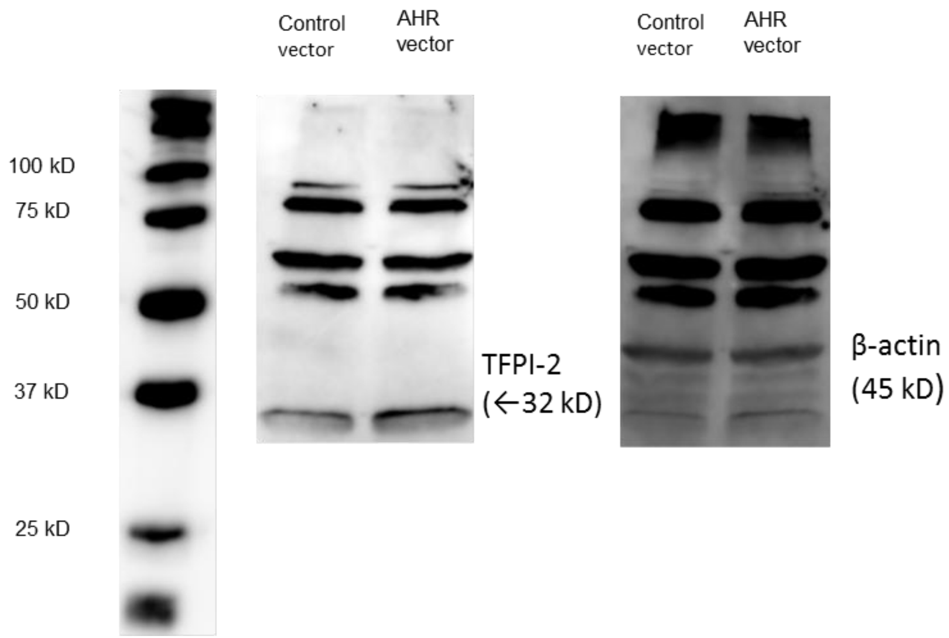
Control vector
AHR vector
Control vector
AHR vector
100 kD
75 kD
50 kD
37 kD
25 kD
TFPI-2
(←32 kD)
β-actin
(45 kD)
